# Potential clinical applications of extracellular vesicles in pancreatic cancer: exploring untapped opportunities from biomarkers to novel therapeutic approaches

**DOI:** 10.20517/evcna.2023.68

**Published:** 2024-05-10

**Authors:** Jose Manuel Sanchez-Manas, Natalia Perez de Gracia, Sonia Perales, Joaquina Martinez-Galan, Carolina Torres, Pedro J. Real

**Affiliations:** ^1^Gene Regulation, Stem Cells & Development lab, GENyO, Centre for Genomics and Oncological Research, Pfizer-University of Granada-Andalusian Regional Government, PTS, Granada 18016, Spain.; ^2^Department of Biochemistry and Molecular Biology I, Faculty of Science, University of Granada, Granada 18071, Spain.; ^3^Instituto de Investigación Biosanitaria ibs.GRANADA, Granada 18012, Spain.; ^4^Department of Medical Oncology, Virgen de las Nieves University Hospital, Granada 18014, Spain.; ^5^Department of Biochemistry and Molecular Biology III and Immunology, Faculty of Medicine, University of Granada, Granada 18016, Spain.; ^#^Authors contributed equally.

**Keywords:** Extracellular vesicles, pancreatic cancer, liquid biopsy, diagnosis biomarkers

## Abstract

Pancreatic cancer is a highly lethal and metastatic malignancy, mainly because it often remains undetected until advanced stages due to the limitations of current diagnostic methods, rendering currently available therapies ineffective. Therefore, it is imperative to identify useful biomarkers for early diagnosis and new therapeutic targets for pancreatic cancer. Recently, extracellular vesicles have emerged as promising biomarkers for the diagnosis and prognosis of pancreatic cancer. Given their presence in various bodily fluids, extracellular vesicles offer a non-invasive approach through liquid biopsy to detect and monitor cancer progression. In this review, we comprehensively examine the multifaceted roles of extracellular vesicles in the progression of cancer, while also exploring their potential as diagnostic, prognostic, and therapeutic biomarkers in the context of pancreatic cancer.

## INTRODUCTION

As of today, cancer is one of the leading causes of death worldwide. In 2020 alone, more than 19 million new cases were diagnosed and almost 10 million people died from cancer. Pancreatic cancer (PC) has become the seventh leading cause of cancer deaths globally, with nearly 500,000 deaths in 2020^[1]^. Mortality data related to PC in Europe in 2020 are indeed alarming. During that year, 132,134 individuals succumbed to PC, representing 29% of the total fatalities due to cancer in Europe. Furthermore, the incidence of PC in Europe is notably elevated, with a rate of 2.31 per 100,000 inhabitants, and a total of 140,116 new cases diagnosed within the same year^[2]^, representing a mortality rate of 94%.

The high mortality rate of PC is largely attributed to the challenges of early diagnosis. It is well-established that PC tends to remain asymptomatic during its early stages or presents symptoms that are often subtle and inconspicuous to patients. Consequently, its detection is frequently delayed until advanced stages, when its highly metastatic nature has led to widespread invasion of the body^[3]^.

Traditionally, PC detection has relied primarily on diagnostic methods involving tissue biopsies^[4]^ and imaging techniques such as Computed Tomography (CT), Magnetic Resonance Imaging (MRI), Positron Emission Tomography (PET), and Endoscopic Ultrasonography (EUS)^[5]^. Nonetheless, these methods come with inherent disadvantages, including high cost, radiation exposure risk, invasiveness, and the potential for disease dissemination. Furthermore, they are time-consuming, not easily repeatable, and do not provide a comprehensive representation of the tumor microenvironment^[4,5]^ or tumor evolution.

To overcome these drawbacks, liquid biopsies have been developed^[4]^. These diagnostic approaches facilitate the identification and monitoring of tumor progression through non-invasive sampling of body fluids such as blood, urine, or saliva. These fluids contain various components that can be harnessed for cancer diagnosis, including circulating tumor cells (CTCs), platelets, circulating tumor DNA (ctDNA), free RNA, and extracellular vesicles (EVs)^[4,6]^.

Among these components, EVs have demonstrated significant potential as diagnostic markers in various cancers, as they serve as key mediators of intercellular communication in both normal and pathological contexts. EVs carry diverse cargo (proteins, lipids, nucleic acids, metabolites, *etc.*) that reflect the state of the cell of origin, which is invaluable for cancer detection^[7,8]^. Specifically, in the context of PC, EVs may contain factors that promote and modulate tumor growth, angiogenesis, metastasis, and chemoresistance^[7]^. Given their pivotal role in PC progression, both EVs and their contents can serve as biomarkers for this pathology.

This review aims to outline diverse methodologies used for the study and characterization of EVs and their role as drivers of tumor development in PC. Additionally, it seeks to assess the potential of EVs as diagnostic, prognostic, and therapeutic biomarkers for PC.

## WHAT ARE EVs?

EVs are nano- or micrometer-sized lipid vesicles secreted by the majority of both healthy and pathological cells^[4]^. This diverse group of vesicles includes various subtypes primarily categorized by their biogenesis and size, namely exosomes (EXOs) (30-150 nm), microvesicles (MVs) (150-1,000 nm), and apoptotic bodies (ApoBDs) (1,000-5,000 nm)^[7]^.

**Figure 1:**
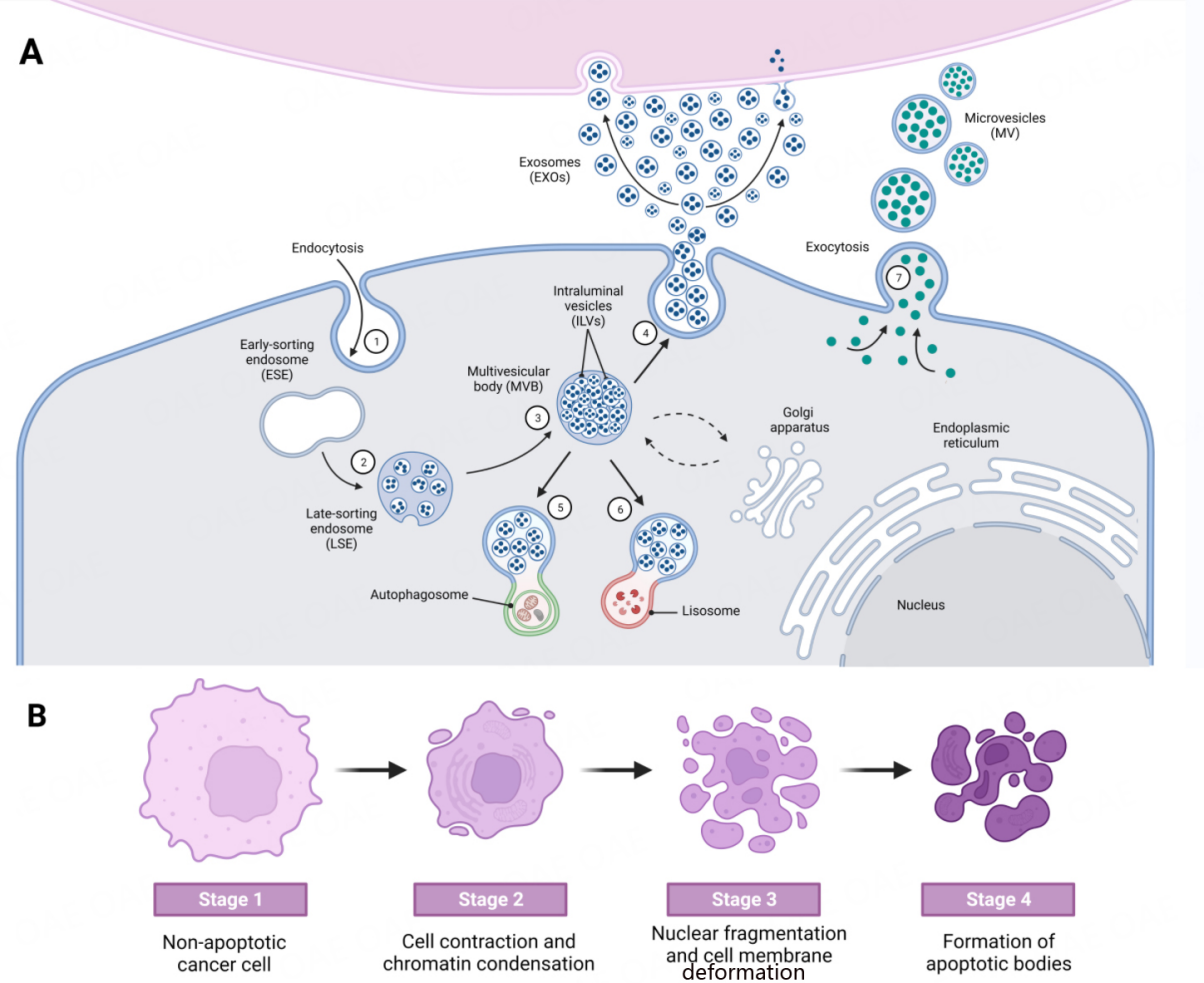
Schematic representation of the biogenesis of EVs. (A) EXOs derive from the endosomal pathway, in which, after a process of membrane invagination, an ESE is formed (1). After maturation, it becomes an LSE (2), which subsequently leads to the formation of an MVB with ILVs or future EXOs inside (3). These MVBs have three possible destinations: fusion with the plasma membrane, releasing the EXOs to the extracellular medium (4); fusion with autophagosomes (5); or fusion with lysosomes (6). MVs derive from the evagination of the plasma membrane (7); (B) Apoptotic bodies are released during the apoptotic process of cells. Figure created with Biorender.

EXOs biogenesis occurs through the endosomal pathway [Figure 1A]. The process involves endocytosis, leading to the internalization of various cargoes, such as lipids, cytoplasmic or membrane proteins, and nucleic acids^[9]^. This internalization results in the formation of early endosomes (ESEs), which then mature into late endosomes (LSEs). These late endosomes generate multivesicular endosomes (MVBs). Following a second process of plasma membrane invagination, MVBs give rise to intraluminal vesicles (ILVs), which will later become EXOs. The fate of MVBs varies, as they can fuse with lysosomes, autophagosomes, or the plasma membrane. In the latter case, exosomes enclosed in MVBs are released into the extracellular medium^[9]^.

The biogenesis of MVs is not as well characterized as that of EXOs. It is known to stem from the evagination of the plasma membrane [Figure 1A]. On the other hand, apoptotic bodies are formed during apoptosis, a programmed cell death process. Similar to MVs, apoptotic bodies are released into the extracellular space through evagination [Figure 1B]^[10]^.

Under normal physiological conditions, all types of cells release EVs into the extracellular space, where they perform various functions, including cellular communication (autocrine, paracrine and/or endocrine signaling) and immune regulation^[4,10]^. As a result, they can be found in different biological fluids such as blood, saliva, and urine^[10]^.

## THE RELATIONSHIP BETWEEN EVs AND PANCREATIC CANCER

As is well known, tumor progression is not directed exclusively by the tumor cells themselves. Numerous cell types play a role in its expansion. The tumor microenvironment (TME) surrounding the tumor comprises fibroblasts, endothelial cells, and cells of the immune system such as lymphocytes, macrophages, and natural killer (NK) cells, among others. These cells primarily function to regulate and impede tumor progression. However, as cancer progresses, the main function of these cells is modified, ultimately contributing to the promotion and expansion of the tumor^[11]^.

**Figure 2:**
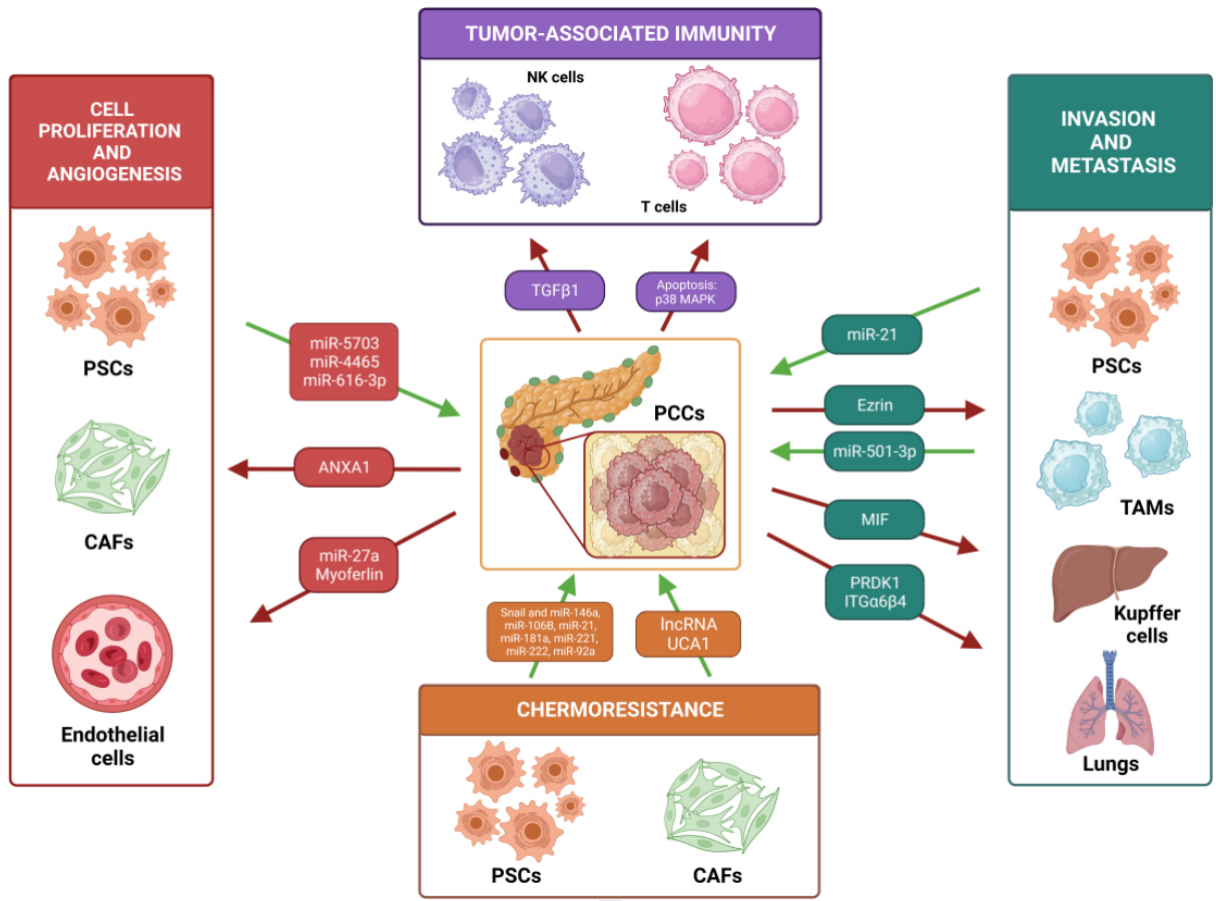
EVs as mediators of the communication between PCCs and TME in PC. The content of EVs derived from PC and from TME cells is capable of modulating PC progression. Pancreatic cancer cells (PCCs), pancreatic stellate cells (PSCs), tumor-associated fibroblasts (CAFs), tumor-associated macrophages (TAMs), NK cells, endothelial cells, and dendritic cells (DCs). Figure created with Biorender.

The most recent pathway by which TME contributes to tumor progression is the communication mediated by EVs. Tumor cells establish a bidirectional communication with TME components through EV secretion. These EVs carry signaling molecules, oncogenic proteins, and nucleic acids, which contribute to tumorigenesis, tumor progression, invasion, and metastasis^[11]^. In PC, the predominant cellular elements constituting the TME include Cancer-Associated Fibroblast (CAFs), Pancreatic Stellate Cells (PSCs), Tumor-Associated Macrophages (TAMs), NK cells, Mesenchymal Stem Cells (MSCs), endothelial cells and Dendritic cells (DCs). Several studies have demonstrated that these TME cells could interact with the Pancreatic cancer cells (PCCs) through a bidirectional communication process mediated by EVs. This interaction is associated with increased tumor growth, tumor progression, angiogenesis, invasion and metastasis, chemoresistance, and tumor immunity [Figure 2]^[7]^.

### Cell proliferation and angiogenesis

As previously mentioned, the communication between PCCs and endothelial cells by EVs can promote tumor proliferation processes and angiogenesis in PC [Table 1]. Among the macromolecules contained in these EVs is myoferlin, a key protein expressed in PCCs-EXOs. This protein is known to be overexpressed in certain cancer types, although it is likewise expressed in EXOs derived from non-tumor cells. The research revealed that the incubation between endothelial cells (HUVEC) with EXOs isolated from PCCs significantly increased the proliferation and migration rates of HUVEC cells. However, when these endothelial cells were exposed to myoferlin-deficient EXOs, their proliferation and migration rates decreased significantly, hence showing a role for myoferlin in PC tumorigenesis^[12]^.

**Table 1:** Role of EVs in PC progression. The content of EVs from PC and TME cells can enhance PC through various pathways: tumor proliferation and angiogenesis, invasion and metastasis processes, chemoresistance and tumor-associated immunity

**Role of EVs in PC**	**Responsible molecule**	**Transmitting cell**	**Receptor cell**	**Action mechanism**	**Reference**
**Tumor proliferation and angiogenesis**	Myoferlin	PCCs	Endothelial cells	ND	[12]
	miR-5703	PSCs	PCCs	Binds to the CMTM4 tumor suppressor gene, inhibiting and activating the PI3K/Akt pathway	[15]
	miR-4465 and miR-616-3p	PSCs	PCCs	Promote the Akt pathway by inhibiting PTEN	[16]
	miR-27a	PCCs	Endothelial cells	Downregulates the tumor suppressor gene BTG2	[14]
	ANXA1	PCCs	CAFs	Interacts with the FRPs receptor and promotes the EMT process	[13]
			Endothelial cells	Interacts with FRPs receptor and promotes the expression of VEGF, αSMA, FAP1α	
**Invasion and metastasis**	Ezrin	PCCs	TAMs	Induces TAMs polarization to M2 phenotype	[18]
	MIF	PCCs	Kupffer cells	Promotes metastatic niche formation by inducing TGF-β release	[17]
	miR-501-3p	TAM	PCCs Lungs and liver cells	Inhibits TGFBR3 tumor suppressor gene	[19]
	PRKD1 ITGα6β4	PCCs	Lungs cells	PRKD1 stimulates the secretion of EVs with ITGα6β4, which promote lung metastasis	[20]
	miR-21	PSCs	PCCs	Induces the EMT process and cell migration through activation of MMP2/9 and the Ras/ERK pathway	[21]
**Chemoresistance**	Snai1 and its target microRNA- 146a	CAF	PCCs	Promote Gemcitabine resistance	[23]
	lncRNA UCA1	PSCs	PCCs	Induces resistance to Gemcitabine via EZH3 recruitment and SOCS3 promoter methylation	[27]
	miR-106b	CAF	PCCs	Promotes Gemcitabine resistance by binding to the 3’UTR of TP53INP1	[25]
	miR-21, miR-181a, miR-221, miR-222 y miR-92a	CAF	PCCs	Inhibition of the expression of the tumor suppressor PTEN and consequent Gemcitabine resistance	[26]
**Tumor-associated immunity**	TGFβ1	PCCs	NK	Decreases the production of cytokines and cytotoxicity factors by NKs	[29]
	ND	PCCs	T cell	Apoptosis induction p38-MAPK- dependent	[28]

ND: not determined; PCC: pancreatic cancer cells; PSC: pancreatic stellate cells, CAF: cancer associated fibroblast; TAM: tumor associated macrophages; NK: natural killer.

Another protein included in PCCs-EVs is ANXA1^[13]^. The effect of ANXA1 and its association with PC were demonstrated by comparing EVs derived from PaCa-2 cells, which contain ANXA1, and EVs derived from KO-ANXA1 PaCa-2 cells. ANXA1 interacted with the Formyl peptide receptors (FPRs) on fibroblasts and endothelial cells. The results demonstrated that migration and invasion processes were more pronounced with ANXA1-EVs than with KO- ANXA1-EVs in fibroblasts, whereas the interaction of ANXA1 with FPRs on endothelial cells led to an increase in the epithelial-mesenchymal transition (EMT). Notably, EMT plays a crucial role in the establishment of the neovasculature during tumor angiogenesis, increasing factors such as Vascular Endothelial Growth Factor (VEGF), Smooth Muscle Actin (αSMA), or Fibroblast Activated Protein 1 (FAP1α)^[13]^.

In addition to proteins, nucleic acids are also involved in the communication between TME and PCCs. Specifically, miR-27a is found to be overexpressed in PCCs-EXOs targeting endothelial cells. miR-27a interacts with and downregulates BTG2, a tumor suppressor gene, promoting the proliferation, invasion and angiogenesis, increasing VEGF expression, in endothelial cells^[14]^.

Conversely, PSCs-EXOs are enriched in miR-5703. miR-5703 has been demonstrated to target the 3’UTR region of the CMTM4 gene, resulting in reduced expression and consequently promoting the proliferation of PCCs. Recently, the role of CMTM4 as a tumor suppressor has been revealed. Overexpression of CMTM4 leads to downregulation of PAK4, inhibiting the PI3K/AKT pathway and the proliferation of PC cell lines^[15]^.

Similarly, it has been evidenced that hypoxia stimulates the overexpression of miR-4465 and miR-616-3p in PSCs-EXOs, targeting PCCs. Both miRNAs specifically recognize PTEN mRNA and decrease its expression. Consequently, the activated AKT pathway promotes the proliferation of PCCs^[16]^.

### Invasion and metastasis

In the same way, EVs and their cargo play a crucial role in triggering invasion and metastasis processes in PC. The liver emerges as the primary metastasis target organ in PC, with a prevalence of 41% compared to 13.9% for lung metastasis^[4]^.

Several factors enhance the process of PC metastasis to the liver [Table 1]. Among them, the macrophage Migration Inhibitor Factor (MIF), overexpressed in PCCs-EXOs, stands out. MIF induces the release of TGF-β by Kupffer cells, leading to the recruitment of bone marrow-derived macrophages and neutrophils to the liver, forming pre-metastatic niches. Furthermore, MIF inhibition has been shown to reduce the process of PC metastasis to the liver^[17]^.

Recent findings demonstrate the role of Erzin, a protein implicated in the regulation of cell proliferation, morphogenesis, migration, and adhesion, in promoting metastasis to the liver. Elevated expression of Erzin in EVs has been correlated with poor prognosis in PC patients. PCCs-EVs enriched with Erzin are linked to the polarization of TAM macrophages into M2 phenotype and contribute to liver metastatic processes^[18]^. M2 macrophages have been associated with pro-tumor activity due to their ability to release anti-inflammatory factors such as TGF-β, which are involved in extracellular matrix remodeling and angiogenesis.

On the other hand, miR-501-3p overexpression in both PCCs and M2 TAM-derived EXOs promotes migration and invasion processes by PCCs, leading to tumor metastasis to liver and lungs in murine models. miR-501-3p inhibits the expression of the TGFBR3 tumor suppressor gene by binding to its 3’UTR region, thereby activating the TGF-β growth factor signaling pathway^[19]^.

It is important to highlight the role of integrin α6β4 (ITGα6β4)-rich PCCs-derived EVs in PC metastasis to the lungs. The low expression of the Protein kinase D1 (PRKD1) in PC promotes the secretion of EVs enriched with ITGα6β4. PRKD1 loss reduces the phosphorylation of its substrate, cortactin, resulting in increased levels of F-actin at the plasma membrane and enhanced secretion of EVs enriched in ITGα6β4 to the lungs^[20]^.

Finally, it has been reported that the existence of EXOs enriched with miR-21 secreted by PSCs. These EXOs induce migration and metastasis processes in PCCs by activating metalloproteinase 2/9 (MMP2/9) activity, which is involved in the EMT process and the Ras/ERK signaling pathway^[21]^.

### Chemoresistance

Drug resistance is a general challenge in PC patients, even with the use of standard drugs such as Gemcitabine, a deoxycytidine analog inhibiting DNA replication and repair^[22]^. However, many PC patients develop resistance to this treatment^[23]^.

The investigation into the origins of this resistance revealed the involvement of the SNAI1 protein and its target, miR-146a. Gemcitabine treatment increased the secretion of SNAI1 and miR-146a-rich EXOs by CAFs. Consequently, these EXOs enhanced the proliferation of the PC epithelial cells, along with resistance to Gemcitabine^[23]^. To address this discovery, GW4869, a sphingomyelinase inhibitor that avoids EXOs formation and release, was employed, leading to a decrease in tumor growth [Table 1]^[24]^. Therefore, these results suggested that the combination of GW4869 with chemotherapy agents could be promising in future *in vivo* trials^[23]^.

Gemcitabine has also been associated with elevated expression of miR-106b in CAFs and CAF-derived EXOs. miR-106b targets the 3'UTR of the *TP53INP1* gene, inhibiting its expression and promoting chemoresistance in PCCs [Table 1]. This gene has also been related to the development of chemoresistance in breast cancer. However, its molecular mechanism of action remains unknown^[25]^.

Continuous Gemcitabine exposure has been correlated to an increase in EXO secretion by CAFs targeting PCCs. Analysis of EXOs content identified five highly overexpressed miRNAs: miR-21, miR-181a, miR-221, miR-222, and miR-92a. These miRNAs collectively inhibit the expression of the tumor suppressor gene *PTEN* in recipient PCCs, thereby promoting chemoresistance and tumor proliferation in PC^[26]^.

The role of PSCs-derived EVs, which are enriched with long non-coding RNA UCA1 (lncRNA UCA1) target PCCs, is noteworthy in the context of Gemcitabine resistance and tumorigenesis. Under hypoxic conditions, lncRNA UCA1 recruits the histone-lysine methyltransferase EZH2 in PCCs, promoting *SOCS3* gene inhibition through promoter methylation. Overexpression of *SOCS3* has been correlated with reduced tumor growth, metastasis, and Gemcitabine resistance in both *in vitro* and *in vivo* studies^[27]^.

### Tumor-associated immunity

EVs play a crucial role in immunosuppression and immune evasion processes in PC. PCCs-derived EXOs have demonstrated the capacity to induce apoptosis in T lymphocytes through the activation of the p38 MAPK-dependent signaling pathway. The activation of this kinase initiates stress activation in the endoplasmic reticulum (ER), triggering the apoptotic mechanism in lymphocytes through the PERK-eIF2α-ATF4-CHOP signaling pathway^[28]^.

On the other hand, EVs derived from PCCs and directed toward NK cells contain elevated TGF-β [Table 1]. Consequently, NK cells reduced the production of cytotoxicity factors and cytokines such as NKG2D, CD107a, IFN-γ, CD71, CD98, and 2-NBDG. This study suggests that the activation of TGFβ1-Smad2/3 signaling pathway is responsible for NK cell dysfunction^[29]^.

## EVs AS DIAGNOSTIC AND PROGNOSTIC MARKERS IN PANCREATIC CANCER

Nowadays, the unique diagnostic biomarker approved by the FDA in PC is CA19-9^[30]^. CA19-9 is a surface carbohydrate highly expressed in PC, which is anchored with an amount of membrane proteins such as mucins or surrounding apolipoproteins^[31]^. Despite its efficacy in detecting PC with a sensitivity of 70%-80%, CA19-9 is somewhat nonspecific because elevated levels are also found in other pathologies such as cholestasis, diabetes mellitus^[30]^, biliary obstructions, pancreatitis, and liver diseases^[31]^.

Furthermore, not all PC patients exhibit elevated levels of this marker^[31]^. Due to its limited specificity, researchers are actively pursuing alternative markers, as well as combinations of markers, to enhance the precision and efficacy of PC diagnosis [Table 2]. The subsequent classification of diagnostic and prognostic markers for PC is based on the nature of the molecule targeted: protein, RNA or DNA.

**Table 2:** Potential markers contained in EVs for the diagnosis and prognosis of PC

**Marker**	**Biological sample**	**Isolation method**	**Detection method**	**Type of study**	**Type of marker**	**Efficiency (%)**	**Ref**
CA19-9 and EGFR	PANC-1 and HPDEC cell lines Plasma	SEC	qSMLM	*In vitro* Preclinical trial	Diagnostic marker	ND	[32]
GPC1	Human cell lines: HMLE, BJ, HDF, HMEL, MCF-7, MDA-MB-231, Panc-1, SW480, HCT-116, T3M4, MIA Paca2 Murine cell lines: NIH/3T3, E10 NMuMG, 4T1, B16F10 Nude (nu/nu) mouse model with previous injections of MDA- MB-231 and MDA- MB-231-CD63GFP cells PKT, KPC and C57BL/6 mouse models Serum	UC and FACS	ELISA and WB	*In vitro* *In vivo* Preclinical trial	Diagnostic marker	AUC = 1 100% sensitivity	[33]
	Plasma	UC	ELISA	Preclinical trial	Diagnostic marker	AUC = 0.59 74% sensitivity	[35]
	Plasma	exoRNeasy Serum/Plasma Maxi Kit	TEM and FACs	Preclinical trial	Prognosis marker	ND	[36]
EphA2	Cell lines: PANC-1, MIA, PaCa-2, BxPC-3, HPNE Nude mouse model (Crl:NU-Foxn1nu) Plasma	nPES	nPES	*In vitro* *In vivo* Preclinical trial	Diagnostic marker	AUC = 0.96 between PC patients and healthy donor AUC = 0.94 between PC patients in early stages and healthy patients	[38]
ZIP4	Cell lines: PC-1.0, PC-1, AsPC-1, Capan-2 Nude mouse model BALB/c Serum	ExoQuick Exosome Precipitation kit	ELISA	*In vitro* *In vivo* Preclinical trial	Diagnostic marker	AUC = 0.8931 between MP patients and healthy donors AUC = 0.89 between MP and BP patients	[39]
HULC	Cell lines: hTERT-HPNE, Panc-1, QGP-1, KP-3, BxPC-3, MiaPaCa-2 Plasma	UC	RT-qPCR	*In vitro* *In vivo* (xenography in nude mice) Preclinical trial	Diagnostic marker	AUC = 0.92	[40]
miR-10b, miR-21, miR-30c, miR-181a y miR-let7a	Plasma	UC y filtración	RT-qPCR	Preclinical trial	Diagnostic marker	AUC = 1 100% sensitivity	[34]
miR451a	Plasma	exoEasy Maxi Kit (Qiagen GmbH, Hilden, Germany)	RT-qPCR	Preclinical trial	Diagnostic marker	AUC = 0.896 between PC patients and healthy donor AUC = 0.855 between PC and BP patients	[41]
*Mutated KRAS*	Cell lines: Panc-1 (CRL-1469) y HMLE Serum	UC	PCR	*In vitro* Preclinical trial	ND	ND	[43]
	Plasma	UC	PCR	Preclinical trial	ND	75.4% sensitivity 92.6% specificity	[44]
*Mutated* *TP53*	Panc-1 (CRL-1469) and HMLE Serum	UC	PCR	*In vitro* Preclinical trial	ND	ND	[43]

MP: Malignant pancreatic cancer; BP: Benign pancreatic cancer; UC: Ultracentrifugation; SEC: Size-exclusion chromatography; FACS: Fluorescenceactivated cell sorting (Flow cytometry); nPES: nanoplasmon-enhanced scattering; qSMLM: quantitative single molecule localization microscopy; WB: Western blot; TEM: Transmission electron microscopy; AUC: Area under the curve; ND: Not determined.

### Protein biomarkers

EVs contain numerous protein-based molecules that have the potential to serve as diagnostic and prognostic markers in PC. A notable example is the epidermal growth factor receptor (EGFR), which has been found to be overexpressed in EVs derived from PCCs^[32]^. Comparative studies analyzing the expression of EGFR and CA19-9 in healthy individuals and PC patients revealed that EVs enriched with EGFR were 35 times more prevalent in PC patients than in healthy individuals. Notably, EVs enriched with CA19-9 were 130 times more abundant in the PC cohort in comparison to the healthy population. However, it is important to note the relatively small sample size in these studies, consisting of 6 healthy volunteers and 5 patients with PC, leading to heterogeneous results within the PC patient group^[32]^.

Another potential diagnostic and prognostic marker is glypican-1 (GPC1), a membrane proteoglycan abundant in EXOs derived from PCCs^[33]^. Elevated levels of GPC1 in EXOs from PCCs have been associated with angiogenesis and metastasis processes in PC^[34]^. Melo *et al.* demonstrated that the presence of circulating GPC1^+^ EXOs (GPC1^+^crEXOs) in the serum of PC patients enables the distinguishment between healthy donors and PC patients, as well as between early or late-stage patients. Furthermore, this study identified the presence of KRAS^G12D^, one of the most frequent mutations in PC patients, within these GPC1^+^crEXOs^[33]^. However, conflicting results were observed in other studies, where the level of GPC1^+^crExo in plasma did not significantly distinguish between healthy individuals and those with PC^[35]^. Notably, Melo *et al.*’s study had a larger cohort (*n* = 246 patients with CP) compared to the present study (*n* = 27 patients with CP)^[33,35]^, potentially influencing the results. Despite this, they concluded that the level of GPC1^+^crExo in plasma enabled them to determine tumor size and distinguish between patients who underwent tumor resection and those who did not. It should be noted that the quantification and detection methodology used by Melo *et al*. might be more complex for clinical reproducibility^[33,35]^.

The potential prognostic value of GPC1^+^ EXOs has also been studied for predicting Advanced Pancreatic Cancer (APC). Plasma levels of GPC1^+^ EXOs were significantly higher in APC patients than in healthy donors. Remarkably, APC patients treated with Regional Intra-Arterial Chemotherapy (RIAC) presented significantly decreased levels of GPC1^+^ EXOs and a higher survival rate. This fact suggests that a low level of GPC1^+^ EXOs could serve as a prognostic marker to predict the outcome of RIAC treatment in PC patients^[36]^.

While research on MVs or large EVs in PDAC remains limited, a recent paper has been published, linking glypican-1 mRNA expression in EXOs and protein levels in tumor-associated MVs, presenting a dual biomarker signature for PDAC. This study sheds light on the potential of utilizing both mRNA and protein expression in distinct extracellular vesicle subpopulations to improve diagnostic and prognostic capabilities for PDAC. This combined analysis effectively discriminates early-stage PDAC patients from those with benign pancreatic diseases and healthy donors across multiple hospitals. Furthermore, among late-stage PDAC patients undergoing chemotherapy, lower levels of GPC1 tMV-mProtein and EXO-mRNA expression before treatment significantly correlate with prolonged overall survival^[37]^.

Eph type A2 receptor (EphA2) is a novel and promising candidate for becoming a PC marker. This receptor, overexpressed in several tumors including pancreatic and esophageal, is associated with tumor progression and metastasis processes^[38]^. Enriched with EphA2, PCCs-derived EVs purified from plasma have demonstrated remarkable capabilities in distinguishing between PC patients and healthy donors, achieving a sensitivity of 94%. In addition, it effectively distinguished between early and late stages with a sensitivity of 91%. Thus, EphA2 outperformed CA19-9 significantly (*P* < 0.001), which had an 81% reliability in discriminating between patients with chronic pancreatitis (CP) and healthy individuals^[38]^.

Another potential diagnostic marker under exploration is ZIP4, a zinc transporter present in plasma cells, endosomes, and EV membranes^[39]^. Increased ZIP4 levels have been linked to the development of several cancers including pancreatic, lung, and hepatic cancers. Jin *et al*. demonstrated, through *in vitro* and *in vivo* assays, that PCCs-derived EVs with high levels of ZIP4 can promote proliferation and migration in PC. Investigating the potential diagnostic value of ZIP4, significant differences in ZIP4 levels were found between patients with malignant pancreatic cancer (MP) and healthy donors, MP and patients with benign pancreatic diseases (BP), and MP and patients with biliary diseases. Furthermore, the diagnostic efficacy of ZIP4 was further confirmed by the area under the curve (AUC) values, which demonstrated its ability to distinguish between MP patients and healthy individuals (AUC = 0.8931), as well as between MP and BP (AUC = 0.89)^[39]^.

### RNA biomarkers

Beyond protein analysis, exploring the RNA content of EVs opens avenues for comprehensive studies. In the context of PC, several RNA types play crucial roles, with a particular focus on lncRNA and miRNA.

#### Long non-coding RNA

LncRNAs are long-stranded non-coding RNAs with more than 200 nucleotides. They commonly have a regulatory role in gene expression and they have been linked to the development of certain pathologies such as PC.

Highly Up-regulated in Liver Cancer (HULC) is englobed among these lncRNAs. Its expression has been linked to the EMT phenomenon. The action of HULC induces phenotypic alterations in PCCs, including the reduction in the expression of E-cadherin, characteristic of epithelial cells, and increased levels of N-cadherin, vimentin, or SNAI1, which are typical of mesenchymal cells^[40]^.

Recently, it has been discovered that TGF-β can induce the EMT process and lead to the appearance and overexpression of HULC in PC cells. Furthermore, PCCs-derived EVs enriched in HULC promote the EMT phenomenon in recipient cells. This induction manifests as increased expression of N-cadherin, vimentin, and SNAI1, thereby enhancing invasion, migration, and tumor growth in the recipient cells^[40]^.

Moreover, the role of HULC as a possible PC diagnostic biomarker has been tested. The results showed its capability to distinguish between PC patients and non-PC patients with an AUC of 0.92. It is important to highlight that non-PC patients included healthy donors and patients with Intraductal Papillary Mucinous Neoplasms (IPMN). Additionally, the study’s sample size was relatively small (*n* = 20 for PC patients), suggesting the need for larger-scale studies to validate its role as a diagnostic biomarker for PC^[40]^.

#### microRNAs

miRNAs are small, non-coding RNAs with the capacity to modulate several physiological and pathological processes, including cancer, by repressing mRNA translation^[34]^. Several miRNAs are notably overexpressed in PCCs-derived EVs. Lai *et al.* identified five miRNAs (miR-10b, miR-21, miR-30c, miR-181a, and miR-let7a) with elevated expression in PCCs-EVs, allowing for a significant differentiation between PC patients and healthy donors, achieving 100% specificity and sensitivity with an AUC of 1. This discrimination is also observed when comparing PC patients with those suffering from CP. The inclusion of CP patients was necessary due to the complexity of distinguishing between both pathologies using diagnostic markers^[34]^.

To assess the reliability of the aforementioned miRNAs compared to GPC1 and CA19-9, the AUC for both markers was estimated. The AUC for GPC1 was 0.75, which was not statistically significant (*P* > 0.05), while CA19-9 showed a sensitivity of 87% and an AUC of 0.92. Furthermore, in contrast to the levels of GPC1-rich EVs, the levels of EXOs rich in these miRNAs decreased significantly 24 h after resection. On the other hand, the levels of CA19-9-rich EXOs were found to be highly variable and thus inconclusive^[34]^. These data collectively suggest that the use of miR-10b, miR-21, miR-30c, miR-181a, and miR-let7a as diagnostic markers is more reliable than the use of GPC1 or CA19-9.

Chen *et al.* recently proposed the use of miR-451a-enriched EVs as a potential diagnostic marker for PC. Their study yielded compelling results, demonstrating that miR-451a is significantly overexpressed in PC patients, effectively differentiating between PC patients (*n* = 191) and healthy individuals (*n* = 90), with an AUC of 0.896. Additionally, miR-451a can distinguish PC patients from those with BP (*n* = 95), with an AUC of 0.855^[41]^.

Furthermore, the levels of miR-451a in serum EVs of PC patients exhibited a positive correlation with the metastatic grade of the tumor. Notably, the levels of miR-451a decreased in patients who underwent chemotherapy or surgery. These findings suggest that miR-451a may serve not only as a diagnostic marker for PC but also as a prognostic indicator, as its levels are sensitive to both the tumor stage and the effects of anticancer therapies^[41]^.

### DNA biomarkers

Numerous gene mutations, including mutations in the *KRAS* oncogene and the *TP53* tumor suppressor gene, have been linked to a positive diagnosis of PC^[42]^. Predominantly, mutations in codons 12 (G12D and G12V) and 13 of the *KRAS* oncogene are the most prevalent. Because KRAS is involved in the RAF/MEK/MAPK and PI3K/PTEN/AKT pathways, these mutations lead to hyperactivated cell division, thereby promoting tumor development and maintenance. Furthermore, KRAS has been implicated in the rewiring of glucose metabolism, as it can enhance glucose uptake, glycolysis, and lactate transport, a phenomenon known as the Warburg effect^[42]^.

Preclinical studies have explored whether the detection of mutations in the *KRAS* and *TP53* genes from EVs can serve as diagnostic markers in PC^[43,44]^. Yang *et al.* confirmed the presence of EXOs with the *KRAS^G12D^* mutation in various groups, including healthy individuals (2.6%), patients with CP (55.6%), individuals with IPMN-type lesions (28.6%), and PC patients (39.6%). While the percentage of patients with *KRAS^G12D^* is higher in those with PC, it is essential to emphasize the relatively low number of individuals in the healthy group (*n* = 9) compared to the larger PC group (*n* = 48). For this reason, it is challenging to use the presence of this mutation as a diagnostic marker for PC, given its presence in all the groups studied^[43]^.

The reduced specificity of *KRAS* mutations for the diagnosis of PC was further underscored in another study analyzing the percentage of mutations in codons 12 and 13 of the *KRAS* gene among individuals with localized PC, locally advanced PC, and metastatic PC. In this study, mutations were observed in all groups, including healthy individuals (7.4%), those with localized PC (66.7%), locally advanced PC (80%), and metastatic PC (85%)^[44]^.

Additionally, the potential role of the mutated *TP53* as a diagnostic marker for PC was also investigated. Specifically, the *TP53^R273H^* mutation was examined in EVs from healthy individuals, patients with CP, individuals with IPMN lesions, and PC patients. The results confirmed the absence of this mutation in the first two groups (healthy individuals and those with PC), while it was present in patients with IPMN lesions (28.6%) and in PC patients (4.2%)^[43]^. However, as before, the sample size may impact the results^[44]^. Therefore, further exploration of the role of *TP53^R273H^* as a predictive marker for PC in EVs necessitates larger-scale clinical studies.

## EVs ISOLATION AND CHARACTERIZATION METHODOLOGIES

**Figure 3:**
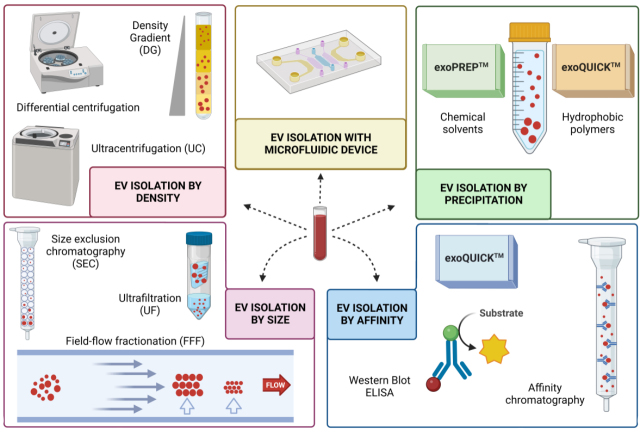
Methods for isolation of EVs from biological fluids. The most commonly used techniques are based on isolation by density (UC, DG); by size (UF, FFF, exclusion chromatography); by precipitation (hydrophilic polymers, organic solvents and commercial kits); by affinity (WB, ELISA, affinity chromatography); and with microfluidic devices. Figure created with Biorender.

To explore the potential of EVs as diagnostic and prognostic markers in PC, it is crucial to understand the various techniques used to isolate and characterize them from biological fluids. Although there’s no standardized method, a diverse range of approaches has been proposed [Figure 3].

Firstly, we have density-based isolation methods such as ultracentrifugation (UC) and density gradient (DG) centrifugation. UC stands out for its reproducibility and ability to handle large sample volumes. Despite being considered a gold standard, it is essential to acknowledge its potential for sample contamination and the need for time-intensive procedures. Nonetheless, combining UC with differential centrifugation offers a promising approach to distinguishing between different types of EVs^[4,45]^.

Similarly, DG centrifugation utilizing iodixanol or sucrose gradients provides an alternative method for EV isolation. Despite sharing some drawbacks with UC, the integration of both techniques has shown promise in reducing protein contamination while maintaining isolation efficiency^[46,47]^.

Moving on to size-based isolation methods, ultrafiltration (UF), Size Exclusion Chromatography (SEC), and Field Flow Fractionation (FFF) offer efficient ways to isolate EVs based on their size. UF is particularly noteworthy for its speed and cost-effectiveness, while SEC preserves EV structure and prevents aggregation. Though each method has its limitations, ongoing advancements continue to refine their efficiency and applicability^[4,6]^.

Moreover, precipitation techniques, such as those utilizing hydrophilic polymers like PEG or organic solvents, provide convenient and cost-effective options for EV isolation. While they may pose challenges such as the risk of contamination, they remain valuable tools, especially for clinical settings^[4,6,47]^.

Commercial kits like ExoQuickTM and ExoPrepTM offer convenient solutions for isolating EVs from clinical samples, although their effectiveness may vary depending on the sample type and specific EV subtypes^[4,6]^. Additionally, techniques leveraging molecule interactions, such as immunoprecipitation assays and affinity chromatography, provide high specificity in EV isolation, particularly when targeting common EV surface markers.

Exciting advancements in EV isolation include novel methods such as the nano-plasmonic exosome sensor (nPES) and heparin affinity assays, offering rapid and sensitive isolation with reduced protein co-isolation^[38,48]^.

Furthermore, microfluidic devices present innovative approaches for EV capture, promising minimal sample volume requirements and automated operation, although further studies are needed to establish their clinical utility definitively^[4,6]^.

After the isolation of EVs has been performed, purification and characterization become imperative to confirm their adequate isolation. The key parameters typically employed for EV characterization are the number, size, concentration, morphology, and markers associated with them^[4]^.

Quantification and characterization of EVs, vital for various biomedical applications, involves a range of innovative techniques. Nanoparticle Tracking Analysis (NTA) and Tunable Resistive Pulse Sensing (TRPS) stand out as effective methods for quantification. NTA, leveraging Brownian motion, offers high accuracy in detecting particles within a wide size range. Although protein contamination can affect precision, NTA provides valuable insights into EV concentrations. On the other hand, TRPS, with its sensitivity, detects particles’ pulses under specific conditions, promising enhanced detection even for smaller EVs^[4]^. Moreover, ongoing advancements in alternative techniques such as nanoscale flow cytometry, surface plasmon resonance (SPR), and surface-enhanced Raman spectroscopy (SERS) hold potential for further improving EV detection efficiency, signaling exciting prospects for future applications^[10]^.

Characterizing EV morphology primarily involves the use of scanning electron microscopy (SEM) and transmission electron microscopy (TEM). While these methods provide valuable insights, it is important to acknowledge that they may influence EV morphology. Nonetheless, they offer detailed visualization, contributing significantly to our understanding of EV structure and composition^[4]^.

Analyzing associated markers, including proteins, nucleic acids, and lipids, offers valuable information on EV composition. Techniques such as enzyme-linked immunosorbent assay (ELISA), Western blot (WB), and polymerase chain reaction (PCR) provide high sensitivity in detecting EV markers, particularly crucial for cancer diagnosis. Despite challenges in lipid marker detection, mass spectrometry (MS) emerges as a promising method, highlighting the continuous advancement in EV characterization techniques^[4,10]^.

Overall, the development and refinement of these methodologies underscore a growing momentum in EV research, offering enhanced precision, sensitivity, and efficiency in characterizing these essential cellular components. To maximize the potential of these techniques, guidelines or recommendations have been established by the International Society for Extracellular Vesicles (ISEV) with experimental approaches to address some of the remaining challenges and to provide robust EV characterization (MISEV 2023)^[49]^.

## EVs AS THERAPEUTIC AGENTS

The utility of EVs extends beyond diagnosis and prognosis; they are also emerging as potent tools in therapeutics applications. The aim is to take advantage of them as natural carrier systems due to their role in cellular communication during both physiological and pathological processes^[50]^. Recent studies have shown that EVs offer several advantages over other *in vivo* therapy delivery methods, such as liposomes or nanoparticles, making them highly promising for therapeutic purposes^[51,52]^.

Highlighting among these advantages is their extraordinary *in vivo* stability, attributed to their natural composition and reduced immunological clearance^[53]^. In fact, several studies have demonstrated that repeated administration of EXOs does not cause signs of toxicity or immunogenicity. Their high delivery efficiency is another strength, with the ability to bind specifically to target cells or tissues via receptors, facilitating controlled release into the corresponding target structures^[53]^. Characterized by excellent transcellular permeability and a variety of release mechanisms including phagocytosis, macropinocytosis, interactions with lipid rafts, caveolae, receptor-mediated endocytosis, clathrin interactions and direct fusion, EVs offer versatile options for therapeutic delivery^[54]^. Additionally, their ability to traverse the blood-brain barrier, thanks to their small size and biocompatible structure, positions them as valuable carriers, especially considering the limitations most drugs face in penetrating the brain^[53]^.

A particularly promising aspect is the potential for highly targeted therapies. EVs from different cell types exhibit an inherent affinity for specific recipient cells, enhancing drug concentration in particular tissues, such as tumors or specific organs^[55]^. This targeted delivery approach allows for a reduction in drug dosage, minimizing toxicity in non-target organs. This phenomenon is influenced by the tissue tropism of EVs, which depends on the expression of specific adhesion molecules. Recent research indicates that alterations in the abundance and identity of tetraspanins and integrins on EVs contribute to changes in molecular interactions with recipient cells, thereby influencing their *in vivo* functions^[56]^.

Another crucial factor in this regard is the “homing” phenomenon, wherein cells exhibit a preference for targeting tissues harboring their progenitor cells, which is often observed in autologous EVs. Recent studies have shed light on this phenomenon, noting that small EVs expressing tetraspanin-8 display enhanced uptake by the pancreas and lungs, in contrast to the liver and intestine^[56]^. While the precise mechanisms controlling tropisms in pancreas are not yet fully elucidated, it has been suggested that an increase in KRAS-induced oncogenic macropinocytosis in PC may play a pivotal role^[57,58]^. Furthermore, the possibility of modifying the EV surface through genetic engineering, including the addition of specific peptide ligands to bind to particular tissues or cells, is underway^[59]^. Techniques are also being developed to load EXOs with specific proteins, such as those containing ubiquitination signals that guide them to the endosomal system and efficiently trap them into EXOs^[60]^.

However, the primary challenge in effectively employing EVs for the treatment of various conditions, including cancer, lies in the standardization of the EV isolation methodologies, the optimization of the cargo amounts due to size variability, and the challenges associated with large-scale production^[52]^. In this context, significant research has been conducted, including studies aiming to generate EXOs and load them with siRNA on a large scale using MSCs in bioreactor systems^[61]^.

Another difficulty in the therapeutic administration of EVs is their rapid clearance from circulation, leading to accumulation in organs such as the liver, spleen, and lungs, as evidenced by previous studies^[54]^. To address these limitations, significant efforts are being made to develop artificial EXOs^[62]^ or to modify them to extend their half-life in circulation^[63]^.

Currently, several potential applications of EVs in the treatment of PC have been proposed, focusing on critical aspects of this disease [Table 3]^[62]^. These include: inhibiting tumor growth, overcoming drug resistance and chemoresistance, modifying the tumor immune microenvironment and targeting mutation-specific therapies (such as those related to *KRAS* gene)^[63-66]^.

**Table 3:** Notable examples of EVs preclinical approaches in PC: different therapeutic approaches with EVs and their main effects on pancreatic cancer both *in vivo* and *in vitro*

**Treatment strategies** **with EVs in PC**	**Responsible molecule**	**Outcome**	**Ref.**
Block EVs release	GW4869	Inhibition of exosome production and decreased drug resistance	[66,67]
Block EVs entry	αvβ3 integrin inhibitor	Inhibiting EVs adhesion	[68]
	shRNA- caveolin-1, flotillin-1, clathrin dynamin-2	Inhibiting endocytosis	[62,69]
Loading chemotherapeutic agents	Paclitaxel	Antiproliferative and tumor regression	[62,70,71]
	Gemcitabine		
Loading miRNA Inhibitor	miR-1231, miR-126-3p, miR- 145-5p, miR-501-3p, miR-27a and miR-125b-5p14	Reducing tumor growth, acting on proliferation, apoptosis, migration, invasion, growth velocity and angiogenesis	[60,62,72]
Overcoming drug resistance	Inhibitor: miR-155, miR-146b, miR-106b, miR-365 and miR- 210	Gemcitabine resistance	[62,73]
	Survivin-T34A or EphA2	Increase drug sensitivity	[75]
Modifying tumor immune microenvironment	STING agonists	Reactivate the immune response	[76,77]
	Inhibiting miR-301a-3p; miR- 155 and miR-125b-2	Macrophage conversion phenotype	[64]
Mutation-specific therapies	siRNA KRAS-G12D	Decrease in tumor size Preclinical Trial	[75]

EVs: extracellular vesicles; PC: Pancreatic cancer.

Since EVs play a crucial role in tumor progression, chemotherapy resistance, and metastasis formation, among others, inhibiting tumor cell-to-tumor cell communication via EVs is emerging as a potential solution against PC. Strategies are being explored to block both the release of EVs, as mentioned earlier with the use of GW4869^[66,67]^, and to interfere with the entry of these EVs into recipient cells by modifying or blocking adhesion and recognition molecules between EVs and target cells^[68,69]^. This approach holds promise for preventing metastasis, given the involvement of EVs in the preparation of the metastatic niche.

Furthermore, EVs are perceived as promising vehicles for loading drugs and therapeutic molecules, taking advantage of their function as natural messengers^[70]^. In this context, the strategy of introducing chemotherapeutic agents into EVs is being developed, aiming to combat the tumor from within^[70]^. Paclitaxel, a drug affecting the cytoskeleton and causing mitotic arrest of tumor cells, has been successfully employed in this approach^[71]^. Recent studies demonstrate that MSC-derived EVs can package and release paclitaxel, resulting in an anticancer effect with potent antiproliferative activity in human PCCs^[70]^. Similarly, investigations involving heterologous EXOs derived from MSCs loaded with gemcitabine or paclitaxel have demonstrated *in vitro* effects on both pancreatic cell lines and MiaPaCa-2 cell xenografts. Additional studies using autologous exosomes generated from the PC cell line Panc-1, loaded with gemcitabine and evaluated on both the cell lines and xenograft models have shown similarly promising results. These studies highlight the effectiveness of drug delivery through EVs, surpassing conventional drug delivery by demonstrating the capability to induce tumor regression in nude mouse models^[62]^.

In addition, EVs offer a versatile platform for loading various therapeutic molecules for PC treatment: miRNAs, small interfering RNAs (siRNAs), and hairpin RNAs (shRNAs)^[52,72]^. Their effects included reducing tumor growth, acting on proliferation, apoptosis, migration, invasion, growth velocity, and angiogenesis^[72]^. Studies in this field have used EVs loaded with miRNA inhibitors, both from MSCs and macrophages (heterologous) and autologous PC lines, demonstrating their impact in PC cell lines and xenograft models. Notable examples of miRNA-loaded EVs for the treatment of PC include miR-1231, miR-126-3p, miR-145-5p, miR-501-3p, miR-27a, and miR-125b-5p14^[62]^.

Regarding drug resistance, there is increasing evidence that EXOs play a crucial role in actively transporting chemotherapeutic agents out of cancer cells, either directly or indirectly^[73]^. Studies involving EVs loaded with inhibitors of resistance-related miRNAs, synthesized by both resistant PC cells themselves and cells in the tumor microenvironment, such as CAFs or macrophages, have been pursued. Strategies include inhibition of miR-155, miR-146b, miR-106b, miR-365, and miR-210, all linked to gemcitabine resistance^[62]^. Another approach explores combining gemcitabine in EVs with miRNA or molecules that decrease resistance generation, such as Survivin-T34A or EphA2, to increase drug sensitivity^[74]^.

Another therapeutic method for PC involves leveraging siRNA targeting mutations in the *KRAS* gene, particularly the extensively studied *KRAS^G12D^* mutation. EVs designed to carry siRNAs specific against *KRAS^G12D^* have demonstrated efficacy, leading to a decrease in tumor size^[74]^. This innovative approach has progressed to a phase I clinical trial, currently under development, holding promise for positive outcomes^[75]^.

Modifying the immune microenvironment is another crucial strategy in PC treatment^[7]^. In the early stages, the immune system activates innate and adaptive immune responses against PC tumor cells^[76]^. However, immune pressure leads to phenotypic selection of PCCs, resulting in a loss of their immunogenicity and the induction of immune tolerance, a phenomenon known as tumor immunoediting. Strategies to reactivate the immune response in PC include the use of immunomodulators such as STING agonists (activators of the interferon gene stimulator pathway) in immunotherapy^[76]^. Studies with PC-derived EVs have revealed the antitumor effect of these agonists when administered via EVs, associated with an increase in proliferative CD8^+^ T cells^[77]^. Inhibiting miR-301a-3p secreted by PCCs suppresses macrophage conversion to the M2 phenotype. Notably, EXOs loaded with miR-155 and miR-125b-2 have been observed to reverse the M2 phenotype to M1, thus stimulating the immune system^[64]^.

## CONCLUSIONS

PC presents one of the most challenging scenarios in oncology due to its aggressive nature, late diagnosis, and limited treatment options. However, recent advancements in the field of EVs offer new hope in the diagnosis, prognosis, and therapy of this deadly disease.

EVs are small membrane-bound vesicles secreted by various cell types, including cancer cells. They carry a cargo of proteins, nucleic acids, and lipids that reflect the physiological state of the originating cells. In PC, EVs play crucial roles in tumor progression, metastasis, and drug resistance, making them promising candidates for use as diagnostic and prognostic biomarkers.

One of the most significant advantages of EVs is their ability to circulate in bodily fluids, such as blood, urine, and saliva, making them accessible for non-invasive sampling. Studies have shown that EVs isolated from the bodily fluids of PC patients contain specific biomolecules, such as microRNAs, proteins, and DNA mutations, which can serve as diagnostic markers for early detection. These biomarkers hold the potential to revolutionize PC diagnosis by enabling clinicians to identify the disease at earlier stages when treatment options are more effective.

Furthermore, EVs show promise as prognostic markers, providing valuable information about tumor aggressiveness, treatment response, and patient outcomes. By analyzing the molecular cargo of EVs, researchers can identify signatures associated with disease progression and predict patient survival, allowing for personalized treatment strategies.

Beyond diagnosis and prognosis, EVs hold immense therapeutic potential for PC. They can be engineered to deliver therapeutic agents directly to tumor cells, bypassing the challenges associated with traditional drug delivery methods. EV-based therapies offer several advantages, including enhanced drug stability, targeted delivery, and reduced systemic toxicity. Additionally, EVs can modulate the tumor microenvironment, inhibit tumor growth, and sensitize cancer cells to chemotherapy, providing new avenues for combination therapies.

In conclusion, the emerging field of EV research offers exciting opportunities for improving the diagnosis, prognosis, and therapy of pancreatic cancer. By harnessing the unique properties of EVs, researchers and clinicians are paving the way for more effective and personalized approaches to combat this devastating disease. As we continue to unravel the mysteries of EV biology and develop innovative EV-based technologies, we move closer to achieving our ultimate goal of improving outcomes for PC patients. It is urgent to make the scientific community aware of the need to achieve consensus and methodological standards in order to get the maximum benefit from this impressive tool.

## DECLARATIONS

### Copyright

© The Author(s) 2024.
